# Development of a sensitive novel diagnostic kit for the highly pathogenic avian influenza A (H5N1) virus

**DOI:** 10.1186/1471-2334-14-362

**Published:** 2014-07-03

**Authors:** Yasuko Tsunetsugu-Yokota, Kengo Nishimura, Shuhei Misawa, Mie Kobayashi-Ishihara, Hitoshi Takahashi, Ikuyo Takayama, Kazuo Ohnishi, Shigeyuki Itamura, Hang LK Nguyen, Mai TQ Le, Giang T Dang, Long T Nguyen, Masato Tashiro, Tsutomu Kageyama

**Affiliations:** 1 Department of Immunology, National Institute of Infectious Diseases, Shinjuku Tokyo 162-8640, Japan; 2 Department of Medical Technology, School of Human Sciences, Tokyo University of Technology, 5-23-22 Nishi-Kamata, Ohta-ku Tokyo 144-8535, Japan; 3 Tsuruga Institute of Biotechnology, Toyobo, Co., Ltd., Fukui 914-8550, Japan; 4 National Institute of Infectious Diseases, Influenza Virus Research Center, Musashimurayama Tokyo 208-0011, Japan; 5 Department of Virology, National Institute of Hygiene and Epidemiology, Hanoi, Vietnam; 6 Pasteur Institute, National Influenza Center, Ho Chi Minh City, Vietnam

**Keywords:** H5 hemagglutinin, Highly pathogenic avian influenza virus, Rapid influenza diagnosis, Monoclonal antibody, Clinical specimens

## Abstract

**Background:**

Sporadic emergence of the highly pathogenic avian influenza (HPAI) H5N1 virus infection in humans is a serious concern because of the potential for a pandemic. Conventional or quantitative RT-PCR is the standard laboratory test to detect viral influenza infections. However, this technology requires well-equipped laboratories and highly trained personnel. A rapid, sensitive, and specific alternative screening method is needed.

**Methods:**

By a luminescence-linked enzyme immunoassay, we have developed a H5N1 HPAI virus detection kit using anti-H5 hemagglutinin monoclonal antibodies in combination with the detection of a universal NP antigen of the type A influenza virus. The process takes 15 minutes by use of the fully automated luminescence analyzer, POCube.

**Resutls:**

We tested this H5/A kit using 19 clinical specimens from 13 patients stored in Vietnam who were infected with clade 1.1 or clade 2.3.4 H5N1 HPAI virus. Approximately 80% of clinical specimens were H5-positive using the POCube system, whereas only 10% of the H5-positive samples were detected as influenza A-positive by an immunochromatography-based rapid diagnostic kit.

**Conclusions:**

This novel H5/A kit using POCube is served as a rapid and sensitive screening test for H5N1 HPAI virus infection in humans.

## Background

Influenza viruses belong to the *Orthomyxovirus* family, whose genome is composed of eight segments of negative-sense RNA encoding 12 proteins. Two major glycoproteins, hemagglutinin (HA) and neuraminidase (NA), are located on the viral envelope, and 16 HA subtypes and 9 NA subtypes of avian influenza A have been identified on the basis of their antigenicities [1,2]. There are three types of influenza virus, namely, A, B, and C. Influenza A (both H1N1 and H3N2 subtypes) and influenza B viruses circulate among the human population each year and are the causative agents of seasonal flu. There have been several pandemics of influenza A infections, which have resulted in the deaths of many humans and animals [2,3]. The high variability of influenza A viruses is driven by frequent mutations in genomic RNA (drift) and by genetic reassortment among avian, porcine, and human strains [4]. This has hampered the development of a universal cross-protective flu vaccine.

The 2003 and 2004 outbreaks of the highly pathogenic avian influenza (HPAI) virus of subtype H5N1 that occurred in poultry and wild birds were genetically traced back to the H5N1 HPAI virus that caused the first outbreak in Hong Kong in 1997, and also those of 2001 and 2002 [5]. This report stated that although the H5N1 HPAI virus remained endemic to that region, it had the potential to become pandemic. The H5N1 HPAI virus has occasionally crossed the species barrier to humans in Asia, resulting in human fatalities [6,7], the first of which was recorded in Vietnam in December 2003 [8]. Numerous clinical cases of H5N1 HPAI virus infections have since been reported in Vietnam, and the disease has spread to other countries in Southeast Asia and the Middle East such as Indonesia, Cambodia, Thailand, Egypt, and Turkey [7,9].

The H5N1 HPAI virus has evolved into many phylogenetically distinct clades and subclades, and these diverse lineages have been largely geographically separated since 2005 [9]. During 2007 in northern Vietnam, the clade 1 virus was displaced by the clade 2.3.4 strain that has a different antiviral susceptibility profile [10,11]. The diversity of this virus is continuing to expand [3,12,13]. Although the earlier endemic outbreak of the avian H5N1 HPAI virus appears to be under control, the threat of a human influenza pandemic remains.

PCR-based molecular tests are one of the most sensitive ways to detect the influenza virus, and conventional and real-time RT-PCR methods have been developed to diagnose H5N1 HPAI virus infections in humans [14-16]. However, only centralized and well-equipped laboratories with trained personnel can perform these analyses. Viral antigen detection using antibodies (Abs) offers an easier and quicker diagnostic test; however, commercially available rapid detection kits for influenza A and B have poor clinical sensitivity for the identification of H5N1 HPAI infection [9]. Using previously prepared monoclonal antibodies (mAbs) against influenza A virus HA of the H5 subtype [17], we developed a rapid, sensitive, and H5N1 HPAI virus-specific diagnostic test kit for H5 HA in combination with detection of the universal NP antigen of type A influenza (H5/A kit), which is processed by a compact and fully automated luminescence analyzer, POCube (Toyobo Co Ltd., Osaka, Japan). In this study, we evaluated this novel H5/A diagnostic kit using clinical specimens infected with the H5N1 HPAI virus (genetically confirmed) in Vietnam and demonstrated the sensitive dual detection of H5 HA and type A nucleoprotein (NP) antigens for the first time. Despite of a limited number of available H5N1 clinical specimens, our results strongly suggest that this diagnostic test is a useful tool in the rapid and reliable identification of H5N1 HPAI virus infections.

## Methods

### POCube system

**Figure 1:**
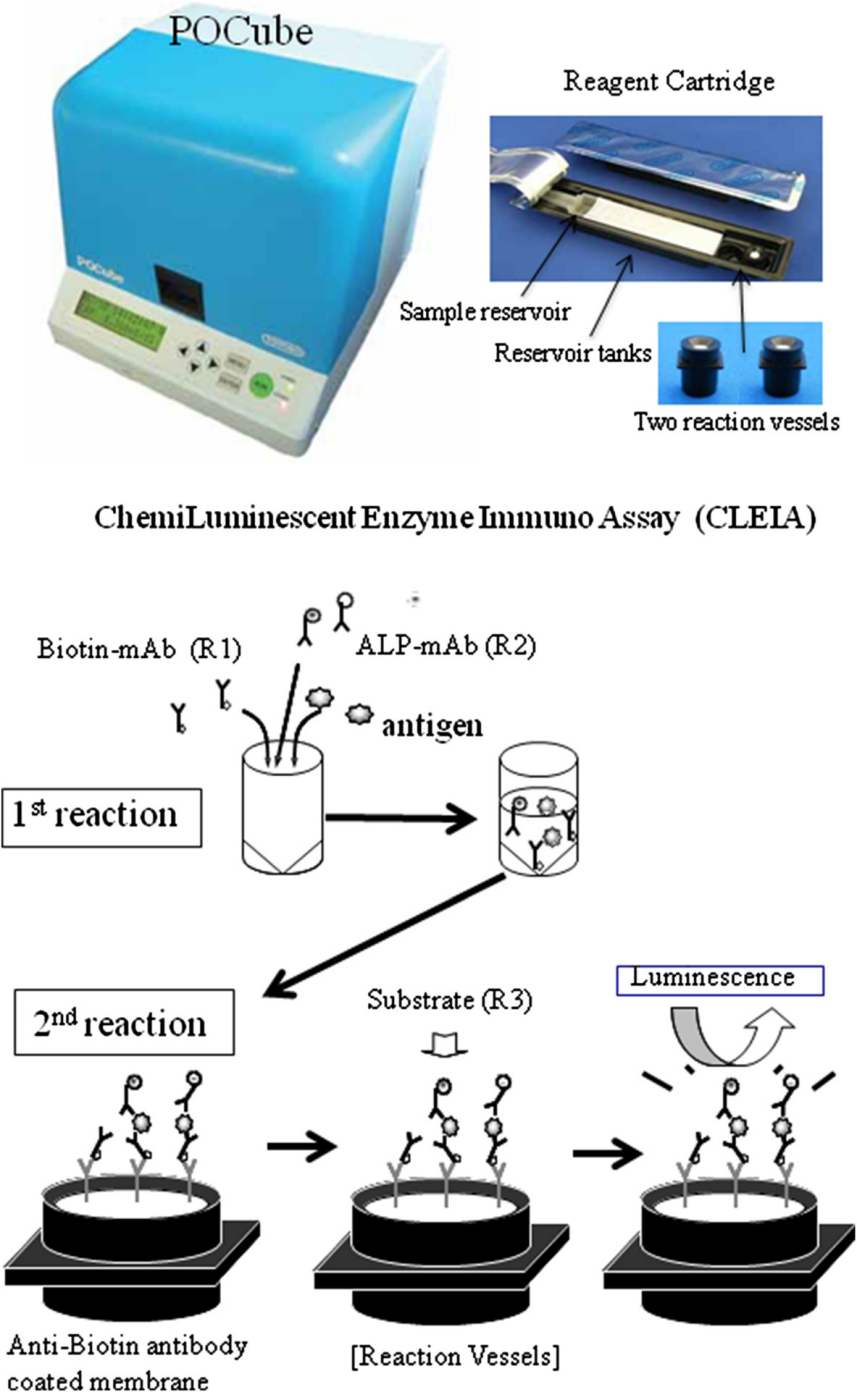
**Principle of the POCube system.** POCube is a fully automated chemiluminescence analyzer. In the H5/A kit, two Abs were incorporated, namely, one for H5 HA and the other for type A NP antigens of the influenza virus. One of the Abs is biotinylated and the other is conjugated to ALP. A sample is loaded into a reservoir tank in the cartridge, which is placed in the machine along with two reaction vessels. When the complex of antigen and two Abs is formed, it is trapped on an anti-biotin Ab-coated membrane in the reaction vessels. The membrane is then washed, a luminescent substrate for ALP is added, and the emitted luminescence is measured. Using the appropriate program, POCube automatically performs each of these steps, and the result is displayed as either positive or negative. Alternatively, the luminescence counts can be provided, if preferred.

The POCube is a fully automated and compact immunological analyzer developed by Toyobo Co. Ltd, which is intended to support the Point-of-Care Testing (POCT) system in clinics. The POCube analyzer is relatively small (280 × 310 × 275 mm), easy to operate, and rapidly measures antigen-Ab complexes by the sensitive detection of luminescence (Figure 1). The POCube system has been used in clinics to detect C-reactive protein (CRP), prostate antigen, influenza A and B viruses, respiratory syncytial virus, in combination with kits authorized by the Ministry of Health, Labour and Welfare in Japan. The principle of the POCube system is to use two mAbs and/or polyclonal Abs that have distinct specificities, where one is biotinylated and the other is conjugated to alkaline phosphatase (ALP). The immune complexes are trapped by an anti-biotin Ab-coated filter membrane in a reaction vessel and the activity of ALP is measured by luminescence output. The whole process takes 5–15 min (depending on the kit), the result is expressed as a luminescence count, and a clinical diagnosis is indicated as “positive” or “negative”.The H5/A kit consists of one cartridge containing two reaction vessels with an anti-biotin Ab-coated filter membrane and following solutions in each compartment of the reservoir tank sealed with an aluminum sheet (Figure 1); a biotinylated anti-H5 HA mAb and a ALP-conjugated anti-H5 HA mAb solutions to detect H5 HA antigens, a commercially available biotinylated and ALP-conjugated mAb set against the type A influenza NP antigen, washing buffer, and a luminescent substrate (APS-5, Lumigen, Inc., Southfield, MI). Up to 180 μl of sample is placed into the first empty reservoir tank within the cartridge. When the POCube operation is started, the sample solution is transferred to reservoir tanks containing each set of Abs and allowed to react at 40°C before the immune complex consisting of antigen, biotinylated Ab and ALP-conjugated Ab is formed. Each reaction solution is then transferred to each vessel and trapped on an anti-biotin Ab-coated filter membrane. The filter membrane is washed with buffer in the tank, substrate is added to each vessel, and the chemiluminescence is measured. The program is set to indicate a positive or negative result for H5 HA and type A influenza virus antigens based on a predetermined cut-off index set that is calculated by adding four standard deviations (SDs) to the average of eight measurements for the negative control. The luminescence counts for eight negative controls in the lot used to test clinical specimens from Vietnam were 1939–2717 (average, 2242; SD, 292) for H5 HA and 1779–2354 (average, 2027; SD, 221) for type A influenza. Thus, the cut-off indices were set at 3410 and 2911, respectively.

The sensitivity of the kit was compared with that of the ESPLINE® Influenza A&B-N (Fujirebio, Inc., Tokyo, Japan) kit, which is a commercially available rapid influenza diagnostic test (RIDT). This immunochromatography (IC)-based kit is meant to diagnose infection by all type A influenza viruses, including H5N1. However, because this kit uses an NP-specific antibody, it cannot discriminate H5N1 from other subtypes.

### Virus preparation

The A/Vietnam/1194/2004 (NIBRG-14) virus, which has a modified HA gene and an NA gene derived from the HPAI A/Vietnam/1194/2004 (H5N1) virus within the backbone of six other internal genes of A/Puerto Rico/8/34 (PR8), as well as other modified H5N1 viruses, A/Indonesia/5/2005 (Indo5/PR-8-RG2), A/turkey/Turkey/1/2005 (NIBRG-23), and A/Anhui/01/2005 (Anhui01/PR8-RG5), were provided by the National Institute for Biological Standards and Controls (Potters Bar, UK). The following subtypes of influenza A viruses were also used to evaluate the specificity of the POCube H5/A kit and were obtained from the Influenza Virus Research Center of the National Institute of Infectious Diseases (NIID), Tokyo, Japan: A/duck/Alberta/35/76 (H1N1), A/duck/Germany/1215/73 (H2N3), A/duck/Ukraine/1/63 (H3N8), A/duck/Czechoslovakia/56 (H4N6), A/turkey/Massachusetts/3740/65 (H6N2), A/duck/Hong Kong/301/78 (H7N1), A/turkey/Ontario/6118/68 (H8N4), A/turkey/Wisconsin/1/66 (H9N2), A/chicken/Germany/N/49 (H10N7), A/duck/England/56 (H11N6), A/duck/Alberta/60/76 (H12N5), A/gull/Maryland/704/77 (H13N6), A/mallard/Gurjev/263/82 (H14N5), and A/duck/Australia/341/83 (H15N8). All viruses were propagated in the allantoic cavity of 10-day-old embryonated chicken eggs. The virus titer and RNA copy number of the matrix gene were determined by the 50% tissue culture infective dose (TCID_50_) using Madin–Darby Canine Kidney (MDCK) cells and by quantitative real-time RT-PCR as described previously [18], respectively.

### Clinical samples

Throat swabs or tracheal aspirates were collected at the point of admission from patients with clinical H5N1 virus infections in Northern Vietnam from 2007 to 2010. Clinical specimens were diluted in about 2 ml of viral transport medium (VTM) consisting of MEM medium supplemented with 1% of 2.92% L-glutamine, 1% of Penicillin- Streptomycin (10,000U/ml and 10,000 μg/ml, respectively), 1.5% of 37.5% NaHCO_3_ and 0.05% of 250 μg/ml Fungizon (all from Life Technologies, Carlsbad, CA), packed into a cooling box and sent to the National Influenza Center, National Institute of Hygiene and Epidemiology (NIHE) for H5N1 HPAI virus detection by conventional RT-PCR. Clinical specimens (nasal and throat swabs) from Southern Vietnam collected in 2009 and 2012 were diluted in about 2 ml of VTM and sent to the National Influenza Center, Pasteur Institute in Ho Chi Minh City (PI-HCMC) for H5N1 HPAI virus detection by real-time RT-PCR. After a part of clinical specimen was used for RNA extraction, the remaining volume was aliquoted into cryotubes and maintained at −80°C until further analysis. The collection of clinical samples was approved by the institutional review boards of NIHE and PI-HCMC. All nasopharyngeal aspirates from patients, including seasonal and non-influenza, were collected after obtaining patient’s written informed consent.

## Results and discussion

Different combinations of anti-H5 HA mAbs [17] and a pair of biotinylated OM-b and ALP-conjugated 1C10 mAbs were selected for H5 HA detection.

**Figure 2:**
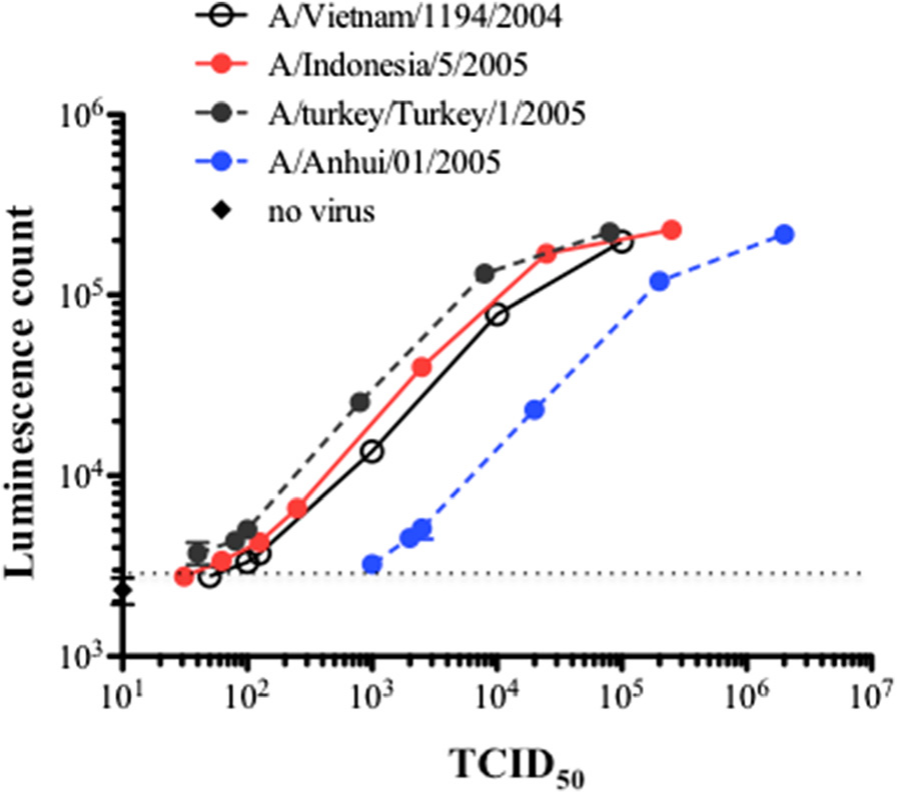
**The sensitivity of the H5/A kit.** H5N1 HPAI virus strains (Table 1) were serially diluted 10-fold, H5 HA antigens were measured using the POCube system, and the luminescence counts for each TCID_50_ virus titer calculated according to each dilution were plotted. The dotted line indicates the lowest detection threshold, which was determined by adding four standard deviations to the average of eight measurements of the negative control.

The H5/A kit contains two sets of mAb mixtures to detect H5 HA simultaneously with NP antigens which are common to all influenza A viruses. This combination is important for the definitive diagnosis of H5 HPAI virus infection. When a clinical specimen is loaded into a reservoir tank in the cartridge, which is placed in the machine along with two reaction vessels, an automatic diagnosis for H5N1 HPAI virus infection is provided (Figure 1). To determine the sensitivity of the H5/A kit, inactivated H5N1 HPAI virus of representative clades (Table 1) were used. The quantities of viruses were determined by TCID_50_ and quantitative real-time RT-PCR targeting the universally conserved matrix gene of influenza A. These viruses were serially 10-fold diluted and the luminescence counts were measured. Then, the virus TCID_50_ titers at each dilution were calculated and plotted as shown in Figure 2. The dotted line indicates the lowest detection threshold, which was determined by adding four standard deviations to the average of eight measurements of the negative control. Thus, the POCube detected these viruses at a titer equivalent to 10^1^–10^3^ TCID_50_.

**Table 1:** H5N1 viruses used for titration

	**Virus strain**	**Clade**	**TCID**_**50**_**/50 μl**	**RNA copies**
			**(Log**_**10**_**)**	**(×10**^**10**^**/ml)**
1	A/Vietnam/1194/2004 (NIBRG-14)	1.1	7.0	2.88
2	A/Indonesia/5/2005 (PR8-IBCDC)	2.1.3.2	7.4	3.81
3	A/turkey/Turkey/1/2005 (NIBRG-23)	2.2.1	5.9	1.60
4	A/Anhui/1/2005 (PR8-IBCDC RG-5)	2.3.4	8.3	3.73

We also confirmed that the kit detected type A antigens, but not H5 antigens, in all other subsets of type A influenza viruses examined (H1N1, H2N3, H3N8, H4N6, H6N2, H7N1 H8N4, H9N2, H10N7, H11N6, H12N5, H13N6, H14N5, and H15N8) including strains recently endemic in Japan (mostly H1N1pdm or type B), indicating that the kit is highly specific to H5 HA (data not shown). Furthermore, we tested clinical swab specimens taken from 15 seasonal influenza and 30 non-influenza patients and found no false positives of H5. Thus, the POCube system offers a simple, safe, and rapid diagnostic system, and is expected to be highly sensitive compared with existing commercial kits that use immunochromatography (IC) technology.

We tested clinical specimens (throat swabs) from nine patients infected with the H5N1 HPAI virus (identification confirmed by molecular diagnosis using RT-PCR) in NIHE during 2007 to 2010 (clade 2.3.4) and compared the detection rate of our H5/A kit by POCube with that of the commercially available rapid influenza diagnostic test (RIDT), (Table 2). The clade was confirmed by genetic analysis of MDCK cells and/or embryonated egg isolation (data not shown). Luminescence counts of POCube were categorized as positive or negative as described in the Methods. In 2007, clinical specimens were collected at two time points from patient A, whereas they were only collected once from each of the other patients (B–I). All the specimens were already diluted approximately 40-fold with VTM. We re-examined these clinical specimens for H5 HA RNA using conventional RT-PCR to assess the quality of the frozen samples: all were positive. One of these 10 samples (sample No. 9 of patient H) was H5-negative but type A-positive (Table 2), indicating that the abundance of viral particles was very low. One sample (No. 2 of patient A) was H5-positive but type A-negative. The quantity of viral particles was lower in this sample (No. 2) than in the sample (No. 1) that was collected at an earlier time point from the same patient (luminescence count of type A was 3,582 in sample No. 2 vs. 122,693 in sample No. 1). By contrast, a widely used commercially available RIDT based on IC using an anti-NP Ab showed very poor sensitivity, with only 10% of samples indicating a positive result. Furthermore, because the influenza NP is highly conserved, this kit cannot discriminate the A/H5N1 subtype from other influenza virus subtypes.

**Table 2:** Detection of H5N1 HPIA virus in clinical specimens in NIHE, Hanoi (clade 2.3.4)

**Year**	**Patient ID**	**Test no.**	**POCube H5/A**^**1**^		**RIDT**^**2**^
			**H5 HA**	**A NP**	
2007	A	1	+	+	+
		2	+	-	-
	B	3	+	+	-
	C	4	+	+	-
	D	5	+	+	+/−^3^
2009	E	6	+	+	-
	F	7	+	+	-
	G	8	+	+	-
2010	H	9	-	+	-
	I	10	+	+	-
% positive			90	90	10

^1^The POCube machine shows the results for H5 and type A viruses as “+” or “–” based on a predetermined cut-off index set (calculated as described in the “Methods”).

^2^RIDT: commercial kit detecting universal NP antigen of influenza A virus (ESPLINE® Influenza A&B-N).

^3^Uncertain.

The H5/A kit was also evaluated using nine clinical specimens collected from one patient (J) in 2009 and from three patients (K–M) in 2012 (Table 3). These specimens were stored at the PI-HCMC after H5N1 HPAI virus (clade 1.1) infection was confirmed by genetic analysis of MDCK cells (data not shown). In patient J, throat swabs taken at two time points, a nasal swab, and a serum sample were all H5-positive and had similar luminescence counts of 5,282, 5,358, 5,344, and 6,448, respectively. In patient L, a throat swab was H5- and type A-negative, whereas a nasal swab was positive for both. In patient M, a throat swab was H5-positive and type A-negative, whereas a nasal swab was H5- and type A-positive. These results suggest that secretion of the virus is more abundant in the nasal mucosa than in the throat, and are not consistent with previous reports [19,20]. Also, the timing for sampling may affect the detection efficiency. However, because the number of samples tested in this study was very small, further testing is needed. The findings indicate that when H5N1 HPAI virus infection is suspected, it is important to collect samples from more than one anatomical location (i.e., throat and nose) and/or at more than one time point.

**Table 3:** Detection of H5N1 HPIA virus in clinical specimens in PI-HCMC (clade 1.1)

**Year**	**Patient ID**	**Test no.**	**Specimen**	**POCube H5/A**		**RIDT**
				**H5 HA**	**A NP**	
2009	J	11	Throat	+	+	-
		12	Throat	+	+	-
		13	Nasal	+	+	-
		14	Serum	+	+	-
2012	K	15	Throat	+	+	+
	L	16	Throat	-	-	-
		17	Nasal	+	+	-
	M	18	Throat	+	-	-
		19	Nasal	+	+	-
		% positive		88.9	77.8	11.1

When samples are H5-positive but type A-negative, such as was the case for patient M (luminescence counts of H5 and type A were 4,071 and 2,622, respectively), the test needs to be repeated or confirmed by genetic analysis. This may occur when the level of virus antigen is at the threshold of detection, such as was the case for sample No. 2 of patient A. Again, the commercially available RIDT showed poor sensitivity, with only one of nine samples testing positive for type A.

Table 4 shows a summary of the results of the POCube H5/A kit test in samples collected from Vietnam, which detected 77.8% and 80.0% of clade 1.1 and clade 2.3.4 H5N1 HPAI virus infections, respectively. In these samples, the POCube test detected 78.9% of H5N1 HPAI virus infections, whereas the RIDT detected only 10.5%. This was probably because POCube is a highly sensitive chemiluminescence detection system that uses mAbs with a high affinity to H5 HA and also analyzes a much larger sample volume than the RIDT (up to 180 μl for POCube vs. about 5 μl for RIDT). Considering that only one in 19 samples tested negative for both H5 and A antigens, the POCube H5/A kit is highly useful to detect H5N1 HPAI virus infections in clinical settings. In this study, samples were diluted in VTM (about 2 ml); however, we believe the detection rate will improved even further if clinical swabs are tested with a reduced volume of this medium.

**Table 4:** Summary of H5N1 HPIA virus detection in clinical specimens from Vietnam

**H5N1**	**POCube H5/A**				**% positive by POCube H5/A kit**	**% positive by RIDT**
	**Positive**	**Negative**	**Suspicious**			
	**+/+**	**−/−**	**+/−**	**−/+**		
Clade 2.3.4	8	0	1	1	80.0	10
Clade 1.1	7	1	1	0	77.8	11.1
Total	15	1	2	1	78.9	10.5

H5 HA HPAI virus detection using POCube and the H5/A kit is a highly sensitive and simple rapid diagnostic system that can be completed within 15 minutes. During the preparation of this manuscript, Sakurai et al. reported a 10–100-fold more sensitive detection of H5 HA using fluorescent beads and an improved IC method [21]; however, the clinical effectiveness of this method remains to be evaluated. We recently determined the epitopes of our anti-H5HA mAbs and found that OM-b, one of mAbs used for the H5/A kit, was broadly reactive to various clades of H5N1 influenza virus isolates in Asia, indicating that ther kit is quite useful for the diagnosis of H5N1 infection in Asian counties [22].

## Conclusions

The POCube system using the H5/A kit is useful for the rapid detection of H5N1 HPAI virus infections in humans in hospitals and other clinical settings where technical resources are limited.

## Abbreviations

HA: Hemmagglutinin; NA: Neuraminidase; NP: Nucleoprotein; HPAI: Highly pathogenic avian influenza; ALP: Alkaline phosphatase; RIDT: Rapid influenza diagnostic test; TCID_50_: 50% tissue culture infectious dose; VTM: Viral transport medium.

## Competing interests

KN and SM are research staffs in Toyobo Co.Ltd. and collaborated with YYY, KO and TK in NIID (a government research institute) for this study, which was formally supported by a grant from the Health Science Foundation of the Ministry of Health, Labor and Welfare of Japan (KHC1218). The patent of H5/A kit was filed by Toyobo Co. Ltd and NIID. This may not cause any competing non-financial interests were they to become public after the publication of this manuscript.

All other authors declare no potential conflicts of interest.

## Authors’ contributions

YTY, KN, SM and KT determined the protocol for the assay. HLKN, MTQL, GTD, LTN prepared clinical specimens and helped YTY and KT for the analysis. MKI, HT and HT participated in the design of the study and helped to draft the manuscript. KO, SI and MT helped to analyze the results and finalize the manuscript. All authors read and approved the final manuscript.

## Authors’ information

YTY worked in NIID for this study and recently moved to the Department of Medical Technology, School of Human Sciences, Tokyo University of Technology, 5-23-22 Nishi-Kamata, Ohta-ku, Tokyo 144–8535, Japan.

## Pre-publication history

The pre-publication history for this paper can be accessed here:

http://www.biomedcentral.com/1471-2334/14/362/prepub

## References

[B1] Kim JK Negovetich NJ Forrest HL Webster RG Ducks: the “Trojan horses” of H5N1 influenza Influenza Other Respir Viruses 2009 3 4 121 128 19627369 10.1111/j.1750-2659.2009.00084.x PMC2749972

[B2] Wright P Neumann G Kawaoka Y Fields Virology, vol. II, 5 edn 2007 Philadelphia, PA: Lippincott Williams & Wilkins, a Wolters Kluwer Business

[B3] Salomon R Webster RG The influenza virus enigma Cell 2009 136 3 402 410 19203576 10.1016/j.cell.2009.01.029 PMC2971533

[B4] Wille M Tolf C Avril A Latorre-Margalef N Wallerstrom S Olsen B Waldenstrom J Frequency and patterns of reassortment in natural influenza A virus infection in a reservoir host Virology 2013 443 1 150 160 23725694 10.1016/j.virol.2013.05.004

[B5] Li KS Guan Y Wang J Smith GJ Xu KM Duan L Rahardjo AP Puthavathana P Buranathai C Nguyen TD Estoepangestie AT Chaisingh A Auewarakul P Long HT Hanh NT Webby RJ Poon LL Chen H Shortridge KF Yuen KY Webster RG Peiris JS Genesis of a highly pathogenic and potentially pandemic H5N1 influenza virus in eastern Asia Nature 2004 430 6996 209 213 15241415 10.1038/nature02746

[B6] Beigel JH Farrar J Han AM Hayden FG Hyer R de Jong MD Lochindarat S Nguyen TK Nguyen TH Tran TH Nicoll A Touch S Yuen KY Avian influenza A (H5N1) infection in humans N Engl J Med 2005 353 13 1374 1385 16192482 10.1056/NEJMra052211

[B7] Kaplan BS Webby RJ The avian and mammalian host range of highly pathogenic avian H5N1 influenza Virus Res 2013 178 1 3 11 24025480 10.1016/j.virusres.2013.09.004 PMC3922066

[B8] Tran TH Nguyen TL Nguyen TD Luong TS Pham PM Nguyen v V Pham TS Vo CD Le TQ Ngo TT Dao BK Le PP Nguyen TT Hoang TL Cao VT Le TG Nguyen DT Le HN Nguyen KT Le HS Le VT Christiane D Tran TT Menno de J Schultsz C Cheng P Lim W Horby P Farrar J Avian influenza A (H5N1) in 10 patients in Vietnam N Engl J Med 2004 350 12 1179 1188 14985470 10.1056/NEJMoa040419

[B9] Abdel-Ghafar AN Chotpitayasunondh T Gao Z Hayden FG Nguyen DH de Jong MD Naghdaliyev A Peiris JS Shindo N Soeroso S Uyeki TM Update on avian influenza A (H5N1) virus infection in humans N Engl J Med 2008 358 3 261 273 18199865 10.1056/NEJMra0707279

[B10] Creanga A Thi Nguyen D Gerloff N Thi Do H Balish A Dang Nguyen H Jang Y Thi Dam V Thor S Jones J Simpson N Shu B Emery S Berman L Nguyen HT Bryant JE Lindstrom S Klimov A Donis RO Davis CT Nguyen T Emergence of multiple clade 2.3.2.1 influenza A (H5N1) virus subgroups in Vietnam and detection of novel reassortants Virology 2013 444 1–2 12 20 23849789 10.1016/j.virol.2013.06.005

[B11] Le MT Wertheim HF Nguyen HD Taylor W Hoang PV Vuong CD Nguyen HL Nguyen HH Nguyen TQ Nguyen TV Van TD Ngoc BT Bui TN Nguyen BG Nguyen LT Luong ST Phan PH Pham HV Nguyen T Fox A Nguyen CV Do HQ Crusat M Farrar J Nguyen HT de Jong MD Horby P Influenza A H5N1 clade 2.3.4 virus with a different antiviral susceptibility profile replaced clade 1 virus in humans in northern Vietnam PLoS One 2008 3 10 e3339 18836532 10.1371/journal.pone.0003339 PMC2556101

[B12] Updated unified nomenclature system for the highly pathogenic H5N1 avian influenza viruses [http://www.who.int/influenza/gisrs_laboratory/h5n1_nomenclature/en]

[B13] Webster RG Govorkova EA H5N1 influenza–continuing evolution and spread N Engl J Med 2006 355 21 2174 2177 17124014 10.1056/NEJMp068205

[B14] Ellis JS Smith JW Braham S Lock M Barlow K Zambon MC Design and validation of an H5 TaqMan real-time one-step reverse transcription-PCR and confirmatory assays for diagnosis and verification of influenza A virus H5 infections in humans J Clin Microbiol 2007 45 5 1535 1543 17473050 10.1128/JCM.02007-06 PMC1865909

[B15] Suwannakarn K Payungporn S Chieochansin T Samransamruajkit R Amonsin A Songserm T Chaisingh A Chamnanpood P Chutinimitkul S Theamboonlers A Poovorawan Y Typing (A/B) and subtyping (H1/H3/H5) of influenza A viruses by multiplex real-time RT-PCR assays J Virol Methods 2008 152 1–2 25 31 18598722 10.1016/j.jviromet.2008.06.002

[B16] Yuen KY Chan PK Peiris M Tsang DN Que TL Shortridge KF Cheung PT To WK Ho ET Sung R Cheng AF Clinical features and rapid viral diagnosis of human disease associated with avian influenza A H5N1 virus Lancet 1998 351 9101 467 471 9482437 10.1016/s0140-6736(98)01182-9

[B17] Ohnishi K Takahashi Y Kono N Nakajima N Mizukoshi F Misawa S Yamamoto T Mitsuki YY Fu S Hirayama N Ohshima M Ato M Kageyama T Odagiri T Tashiro M Kobayashi K Itamura S Tsunetsugu-Yokota Y Newly established monoclonal antibodies for immunological detection of H5N1 influenza virus Jpn J Infect Dis 2012 65 1 19 27 22274153

[B18] Nakauchi M Yasui Y Miyoshi T Minagawa H Tanaka T Tashiro M Kageyama T One-step real-time reverse transcription-PCR assays for detecting and subtyping pandemic influenza A/H1N1 2009, seasonal influenza A/H1N1, and seasonal influenza A/H3N2 viruses J Virol Methods 2011 171 1 156 162 21029748 10.1016/j.jviromet.2010.10.018 PMC7173154

[B19] de Jong MD Simmons CP Thanh TT Hien VM Smith GJ Chau TN Hoang DM Chau NV Khanh TH Dong VC Qui PT Cam BV Ha do Q Guan Y Peiris JS Chinh NT Hien TT Farrar J Fatal outcome of human influenza A (H5N1) is associated with high viral load and hypercytokinemia Nat Med 2006 12 10 1203 1207 16964257 10.1038/nm1477 PMC4333202

[B20] Uyeki TM Human infection with highly pathogenic avian influenza A (H5N1) virus: review of clinical issues Clin Infect Dis 2009 49 2 279 290 19522652 10.1086/600035

[B21] Sakurai A Takayama K Nomura N Munakata T Yamamoto N Tamura T Yamada J Hashimoto M Kuwahara K Sakoda Y Suda Y Kobayashi Y Sakaguchi N Kida H Kohara M Shibasaki F Broad-spectrum detection of h5 subtype influenza a viruses with a new fluorescent immunochromatography system PLoS One 2013 8 11 e76753 24223117 10.1371/journal.pone.0076753 PMC3819354

[B22] Kobayashi-Ishihara M Takahashi H Ohnishi K Nishimura K Terahara K Ato M Itamura S Kageyama T Tsunetsugu-Yokota Y Broad Cross-Reactive Epitopes of the H5N1 Influenza Virus Ide ntified by Murine Antibodies Against the A/Vietnam/1194/2004 Hemagglutinin PLoS One 2014 9 6 e99201 24945805 10.1371/journal.pone.0099201 PMC4063728

